# A Practical Essay on Some of the Principal Surgical Diseases of India

**Published:** 1841-10-01

**Authors:** 


### A Practical Essay on some of the Principal Surgical Diseases of India. By *F. H. Brett*, Esq. M. R. C. S. L. Bengal Medical Service, Surgeon to the Right Honorable the Governor General's Body Guard. Calcutta, 1840.

The plan, an useful one, of this work is to treat of all the *surgical diseases* met with in India. As these must, in the main, resemble those of other portions of the world, we must be excused from going through them systematically. All we can do is to select such passages as contain interest or instruction in regard to diseases incidental to the East.

### *The European Constitution in India.*

An *European* on his arrival in India is generally in the highest state of plethora. By such a system of stimulating diet he seldom escapes a severe attack of inflammation shortly after his arrival. Mr. Brett points out the want of a medical superintendent of Cadets. They commit the most serious errors of diet and of regimen. The consequence is an attack of illness. On convalescence, the cadet renews the same habits, and he arrives in the upper provinces with his health restored. For the first few years of his residence in India he becomes in high condition especially during the cold season. (We allude particularly to officers and civilians.) But his system, both vascular and nervous, is under a constant state of excitement, from the continued in-pouring of nutriment, stimulation and high temperature, and he labours under plethora from *repletion*, as well as plethora from *diminished secretion*. He is on the precipice of disease, and a slight impulse is sufficient to destroy the balance and precipitate him.

Should he survive the repeated attacks to which he is liable his constitution becomes enfeebled, his appetite impaired, his energies diminished, the secretions and excretions scanty, and the skin dark, harsh and dry. He is perhaps dyspeptic, and, in a word, his system becomes lowered down to the climate, and he is no longer recognizable as the energetic active European. His muscular system is much reduced. He is the "dried up Indian," with habits and constitution scarcely adapted for his native land. On the other hand, should his constitution still remain of the plethoric tendency, he is gross, corpulent and flabby.

Mr. Brett eulogises *gymnastics*. If Europeans, he remarks, cannot adapt their mode of life to a tropical climate, but must indulge in habits totally incompatible with such a climate, they should at all events endeavour to convert their food into wholesome nutriment, and preserve the robustness of their frames by practising athletic exercises, in the cool of the day, and wrestling in imitation of the Puh,lwans. It is indisputable that these individuals enjoy an immunity from disease, unknown by others. There are none whose constitutions resist the exciting causes of disease so well, although their blood is abundant and their vascular system vigorous. The few Europeans who have entered thoroughly into the spirit of these exercises return to their native land with vigorous constitutions capable of really enjoying their native country.

The writer has long admired and practised the calisthenic exercising of the Asiatics, and attributes a better state of health and stamina, and a capability for active pursuits, far superior to that enjoyed by him in England, to a systematic use of these exercises.

In the Governor General's Body Guard there is a very good specimen of a gymnasium amongst the troopers, some of whom are very well developed athletics, among the old and most respectable hands.

The gymnasium of the Asiatics much resembles that of the ancient Greeks, first introduced at Lacedæmon. The Hindoos perform these feats in imitation of Ram, the deity of war and victory. They have the "Palæstra" (Ukhara) for wrestling and other feats of agility, the Conistereum or conistra, in which they cover themselves with sand or dust, and the stadium for spectators. There is likewise the gymnasiarcha, (Khuleefa) who is the director and superintendant of the whole elected by general consent for superiority as having vanquished all the rest: the Zystarcha who presides in the Zystus or stadium. What originated in the early establishment of society for the purposes of acquiring strength and dexterity in repelling an enemy, became converted into a part of medical regimen when luxury and idleness reduced them to the sad necessity of applying to physicians, who referred them to the practice of gymnastics as a preservative, as well as a means of re-establishing health.

The "Moogdurs" the "Dund'h" and the "Lézum," are the best kind of exercises for general use in India, though it would be well for a young man to go through the whole system at first under a regular "Puh,lwan," and afterwards continue the Moogdurs, Dundh and Lézum in moderation, as a high degree of artificial training may be carried too far; excess, even in what is good, is to be avoided. Nothing is so conducive to a perfect capillary circulation, to the healthy action of the liver and of all the secretions, the tone of the stomach and the sthenic state of the nervous and muscular system, enabling us to bear up against a long and sultry day. Friction and shampooing should not be omitted.

The Romans carried these athletic exercises to their utmost pitch, accompanied by all the state and magnificence of wealth, but it participated in the general downfall of that empire; and until recently the association of medicine with gymnastics was not renewed in Europe.

Moderate stimulation is requisite to the European in India, water drinkers are not observed to be the healthiest people.

### *Sloughing of the Cornea from imperfect Nutriment.*

Instances of this description, illustrating the celebrated experiments of Majendie, occurred amongst the prisoners in the jails of Mooradabad, Shahjehanpore and Cawnpore. These unhappy creatures were subject in the highest degree to every debilitating cause. Imperfect nourishment, their aliment possessing neither diversity, nor multiplicity of ingredients, impure air, especially confinement at night in closed wards surrounded by high walls, excessive heat in the Summer, severe cold, and great range of temperature in the Winter months, fatigue and mental depression. Many of these were exposed to an endemic dysentery, under which if they did not succumb, they became reduced to the very lowest ebb of debility, torpor and apathy. Almost all the secretions were suspended excepting that of the mucous membrane of the bowels. Their tongues were pallid, their extremities shrunk, and the surface of the whole body cold, even in Summer. There was no cutaneous transpiration.—Their eyes were glassy. Under this the inflammation of the conjunctiva occurred, an ulcer formed on the cornea, which speedily sloughed and penetrated the whole of the layers, followed by an evacuation of the humors of the eye. It was particularly remarked in *all* the cases, that there was *no pain*. There was increased secretion from the Meibomian and lachrymal glands, and suppuration of the conjunctiva. The anterior chamber became filled with a muddy purulent fluid. The ulcer of the cornea sloughed, the lens became evacuated, and the eyeball of course collapsed. Both eyes were often affected, and the patient generally expired in an extreme state of emaciation.

### *Advantages and Disadvantages of Operating for Cataract, when one Eye is Affected.*

Mr. Brett presents a sort of table of the pros and cons of this important question. Our readers may like to glance over them.

*Objections, 1st.*—One eye being sufficient for the purposes of life, why therefore subject the patient to the pain and inconveniences attending the operation ?

*Arguments in favor of Operating.*—1st. When one eye is diseased, blindness must almost necessarily follow by the formation of cataract in the other eye. Ergo, it becomes necessary to anticipate the disease in the second eye, by endeavouring to restore the sight in the eye first attacked.—2nd. Many people have a strong as well as a weak eye, and the former more frequently becomes diseased.—3rd. Obscurity from sympathy and habit, often results from a patient becoming blind of one eye ; especially when first accidentally discovered by him on his closing the sound eye.—4th. The sphere of vision with one eye is considerably less than with two.

*Objection, 2nd.*—Great inconvenience has occasionally resulted from confusion of vision, occasioned by the different refracting power of the two eyes, one possessing and the other not possessing the crystalline humour.

*Arguments in favour of Operating.*—*1st.* Confusion of vision is not always or even generally the result of the operation.—2nd. Supposing that confusion of vision generally did occur, the arguments in answer to the 1st objection would equally apply to this.—3rd. Again, the extreme anxiety of the patient in the anticipation of the disease ensuing in the other is a strong inducement for operating.—4th. The length of time likewise for the patient to wait until he becomes blind in both eyes, seems a needless delay, and painful state of suspense, seeing the period may vary from a few months to many years.—5th. *The diseased eye, favourable for operation, may become unfavourable,* first by accident, secondly, by inflammation of an acute or chronic character, adhesion to the iris, &c. *Amaurosis also sometimes follows from delay,* and change in the consistence and volume of the lens produces sometimes internal inflammation, and also absorption of the vitreous humour *which an early operation might have prevented.*

*Objection 3rd.*—An eye which has undergone an operation with every success, never obtains that perfection of vision which is possessed by a perfectly sound eye.

*Arguments in favor of Operating.*—Such imperfection is remedied by the employment of good glasses ; and the question is not as to the patient being short-sighted, but as to his having any vision at all, seeing that the sound eye becomes generally blind. Moreover, the advantage of vision even with a glass, is preferable to blindness without, in that eye, in every respect.

*Objection, 4th.*—When only one eye is affected, the operation has not such a brilliant effect, and the patient is seldom satisfied.

*Arguments in favor of Operating.*—Such a consideration is not of sufficient weight against the preceding arguments.

A few additional arguments in favor of operating may be adduced, but these are not of universal application, viz. 1st. The patient may have originally laboured under short sightedness (Myopia). Some of these patients see better after the operation than they did before, or than with the other eye.

2nd. Patients of an advanced period of life, who become affected with presbyopia, becoming affected with cataract, often see extremely well after the operation with the use of glasses.

3rd. Beer and others are of opinion that from the great sympathy between the two eyes, the morbid action of the sound eye may be prevented by the removal of the complaint in the diseased eye. But satisfactory cases in illustration have not been adduced. It is contrary, I should say, to general experience.

4th. The patient being young, a soldier, &c. are among the minor inducements for operating.

### *Indian Operation for the Stone.*

The patient was seated on the lap of an assistant, with his back towards the assistant's face. The hands were secured under his hams, the thighs being bent at the acutest possible angle, and the knees likewise.

The operator commenced by kneading the abdomen, with his hands, well lubricated with oil, and pressing the bladder, moderately distended with urine, from the hypogastric region, downwards towards the outlet of the pelvis; and keeping up the pressure with his right hand, he introduced the fore and middle fingers of his left hand into the rectum, as deep as possible, grasping the stone behind the base of the prostate gland, and drawing the stone to the perineum, where it made a distinct projection.

A *packing* needle was then employed, by plunging it into the perineum, striking the stone, and removing all doubt of the presence of the calculus in perineo, before attempting any incision. These efforts were attended with a considerable deal of dragging and distention of the internal parts.

Inclining the patient more towards the horizontal, he now commenced the incision a little to the left of the raphe, making repeated and hesitating cuts. Mr. Brett introduced a sharp scalpel into his hand and induced him to enlarge the incision considerably. When he had so done he passed the hooked extremity of a vectis behind the stone, and by a digging effort, and applying the power to the handle, using the arch of the pubis as the prop, he, by main force, started out the stone, breaking it, however, into a number of pieces; the calculus was soft, of the friable description.

Mr. Brett adds :—

"I endeavoured to mitigate the sufferings of the patient by all the suggestions I could make, and had I not insisted on larger and bolder incisions being made, or allowed the vectis to be introduced until they were completed; or had I not checked his hand when using the latter to force out the stone with great violence, fatal consequences, perhaps, might have ensued. The European instruments were shewn him, and he acknowledged their infinite superiority. A native operator confessed that the average mortality was 40 per cent." 503.

This work will prove extremely useful to our Indian brethren, and more particularly to the natives now brought up as surgeons.

